# Proto-Plasm: parallel language for adaptive and scalable modelling of biosystems

**DOI:** 10.1098/rsta.2008.0076

**Published:** 2008-06-17

**Authors:** Chandrajit Bajaj, Antonio DiCarlo, Alberto Paoluzzi

**Affiliations:** 1 Department of Computer Sciences, Center for Computational Visualization, Institute for Computational Engineering and Sciences201 East 24th Street, ACES 2.324, Austin, TX 78712-0027, USA; 2 Department of Studies on Structures, Modelling & Simulation Lab, Università Roma TreVia Corrado Segre, 6 00146 Roma, Italy; 3 Department of Informatics and Automation, Geometric Computation Lab, Università Roma TreVia della Vasca Navale, 79 00146 Roma, Italy

**Keywords:** computational biology, geometric and physical modelling, multilevel and multiphysics simulation, high-performance computing

## Abstract

This paper discusses the design goals and the first developments of Proto-Plasm, a novel computational environment to produce libraries of executable, combinable and customizable computer models of natural and synthetic biosystems, aiming to provide a supporting framework for predictive understanding of structure and behaviour through multiscale geometric modelling and multiphysics simulations. Admittedly, the Proto-Plasm platform is still in its infancy. Its computational framework—language, model library, integrated development environment and parallel engine—intends to provide patient-specific computational modelling and simulation of organs and biosystem, exploiting novel functionalities resulting from the symbolic combination of parametrized models of parts at various scales. Proto-Plasm may define the model equations, but it is currently focused on the symbolic description of model geometry and on the parallel support of simulations. Conversely, CellML and SBML could be viewed as defining the behavioural functions (the model equations) to be used within a Proto-Plasm program. Here we exemplify the basic functionalities of Proto-Plasm, by constructing a schematic heart model. We also discuss multiscale issues with reference to the geometric and physical modelling of neuromuscular junctions.

## 1. Introduction

The physiome of an individual or a species is the description of its functional behaviour. Nowadays, the term ‘physiome’ also refers to models of human physiology based on mathematical methods and computational techniques, traversing several disciplines (physics, chemistry, biology and bioengineering, medicine, informatics) and diverse spatial and temporal scales (from subcellular to holistic, from nanoseconds to tens of years). The Physiome Project (Higgins *et al.* 2001) was the first worldwide effort to provide a computational framework for understanding human physiology. It aimed to develop integrative models at all levels of biological organization from genes up, via gene regulatory networks, protein pathways, integrative cell functions and structure–function relations for tissues and whole organs. The VPH (virtual physiological human) is a European initiative (Clapworthy *et al.* 2007) intending to provide a unifying architecture for the integration and cooperation of multiscale physiome models. It is largely recognized that evolving physiome activities will increasingly influence medicine and biomedical research, with an ever increasing demand for specific and robust computational platforms.

Field modelling and simulation dominate computational science and engineering. The standard engineering process requires repeated iterations of shape design, simulation, evaluation and feedback. Advances in computer technology, computational science and simulation methods have made such iterations more efficient and accurate, raising the productivity and shortening the time to market. But the iterative process itself has not changed significantly; it involves a pipelined sequence of modelling tasks, computational steps and representation conversions, such as meshing.

Computer simulation entered biology more recently, to help in understanding the basic mechanisms that underlie life on a hierarchy of scales, from proteins to cells to tissues, organs and systems (see Zhang *et al.* 2005*a*; Cheng *et al.* 2007). Here, geometry plays a primary role in determining the behaviour of living components and their interactions within living assemblies, at all scales. Moreover, their geometric configuration cannot be considered as given *a priori*; in general, it has to be computed concurrently, taking into account chemical, electrical and mechanical interactions. These novel application domains are characterized by a huge size of computer models. In particular, novel bioengineering approaches to the challenging VPH project require a strict integration of terascale geometric data, multiphysics simulation, concurrency-based adaptive behaviour and multiscale functional specialization. The massive data size is due to several factors. First of all, simulations require cellular decompositions, instead of more compact boundary representations. This fact alone accounts for one order of magnitude increase in model size. Also, the common factoring of repeated substructures, exploited to a great extent by large-scale hierarchical models in computer graphics, cannot be benefited from when dealing with large deformations, thus producing an increase of several orders of magnitude in the model size. Finally, consider the sheer number and complexity of components: there are thousands of atoms in a protein, several thousand proteins in a cell, and so on.

Consequently, computational modelling of human biosystems has to face great challenges. Our vision is that description languages are not sufficient, and that totally new progressive and adaptive methods for terascale geometric modelling are needed, combined with novel adaptive methods for multiphysics and multiscale simulation, working on parallel and distributed supercomputers. Both symbolic and hierarchical characterizations of the various components should be allowed for, as well as shape reconstruction from high-resolution nuclear magnetic resonance (NMR) and other volume imaging techniques. The ambitious goals of symbolic-driven computational bioscience and real-time interaction with terascale data are not achievable without a computational format capable of combining the geometric and physical representations and algorithms. Our proposal is based on a novel algebraic topological approach to field modelling (DiCarlo *et al.* 2007; Milicchio *et al.* 2008), in which the field problem is formulated directly in terms of a decomposition of its domain into cells of codimension zero, i.e. full dimensional.

It is to be remarked that the computation of complex geometric and solid models is commonly regarded as hard to parallelize. More than a hundred papers could be cited which consider parallel rendering and visualization of both volume and surface geometric models. On the contrary, very few previous attempts to parallel shape generation are to be found in the literature. The paucity of parallel approaches to geometric modelling is due to the extreme complexity of boundary data structures currently used in solid modelling and their lack of implicit space indexing.

Instead, we use a twin representation of geometry and topology, combining binary space partition (BSP) trees (Naylor 1990; Naylor *et al.* 1990), which store no topological information, with a complete representation of the stepwise-generated mesh topology (Bajaj & Pascucci 1996) associated with the Hasse diagram of the polytopal complex (Ziegler 1995). Our design choice allows the model generation to be split into fragments to be distributed to computational nodes for progressive detailing. An algorithm for parallel, progressive Boolean operations is given in Paoluzzi *et al.* (2004); several graphics and modelling techniques are integrated into the same format in Scorzelli *et al.* (in press).

Another significant difference between our approach and more conventional ones is that we focus on solid mesh decomposition, instead of boundary representation. This choice provides us with both a so-called ‘embarrassingly parallel’ native decomposition of the simulation domain, and a native support for simulations that does not require intermediate domain re-meshing.

The rest of the paper is organized as follows. Section 2 introduces our parallel computational environment, putting Proto-Plasm into perspective with respect to the existing data languages for integrative biology. The main features of our computational platform are summarized in §3, and illustrated through some simple examples in §4. Section 5 provides a brief discussion of the multiscale modelling of neuromuscular junctions (NMJs). Section 6 presents a few concluding remarks.

## 2. The computational environment

The Proto-Plasm computational framework will be composed of (i) a specialized generative language, (ii) libraries of parametrizable parts and models, (iii) an IDE (integrated development environment) providing the user with a set of programming and visualization tools, and (iv) a parallel engine able to make use of the available hardware resources.

### (a) Proto-Plasm features

The computational framework is a specialized and high-performance extension of the geometric language Plasm (Paoluzzi *et al.* 1995; Paoluzzi 2003), strongly inspired by the functional language FL (Backus *et al.* 1990). It is currently being embedded in the programming language Erlang (see Armstrong 2007*a*,*b*) for parallel and concurrent execution. Erlang—originally developed by Ericsson for soft real-time support of network switches—seems to fit very well with multicore CPUs and symmetric multiprocessing (SMP) architectures. FL was designed to be a successor to Backus' earlier FP programming language (Backus 1978), capable of providing specific support for what Backus termed *function-level* programming and treating programs as mathematical objects. In the FL style of programming, a program is built from simpler programs, combined by program-forming operations called *functionals*.

Plasm extends FL with a geometric type (hierarchical polyhedral complex) and with native geometric operations, including extraction of skeletons of any dimension, Boolean union, intersection and difference, Cartesian product, and Minkowski sum of polyhedral complexes. The language supports *parametric* geometry by mapping the curvilinear coordinate functions of embedded manifolds—curves, surfaces, solids and higher dimensional objects—from a simplicial decomposition of the domain of a chart. *Implicit* representations (by which geometrical objects are defined as level sets of a field) are being added to the geometric kernel using the *Ganith* libraries (Bajaj 1990), an algebraic toolkit for manipulating polynomials and power series of arbitrary degree. Last but not least, the language is dimension independent by design and geometric validity is syntactically guaranteed.

A Proto-Plasm script is written using only the following three programming constructs: (i) the *application* of a function to its input parameters, providing the output value, (ii) the *composition* of two or more functions, producing the pipelined execution of their reversed sequence, and (iii) the *construction* of a vector function, allowing for the parallel execution of its component functions. A Proto-Plasm script contains a set of *definitions* and one or more *expressions*. A caching of values to be computed is associated to the expression graph, where already evaluated subexpressions are connected via symbol and value sharing. The traversal of the reachable subgraph of the functional environment is activated whenever a new value is being evaluated or an existing symbol is redefined. The value generating process is made easier by the fact that the language is purely functional—no state nor side effects.

Proto-Plasm will support *progressive* computations, where each operation works by reading a stream of incremental refinements of its operands and produces from the very beginning a coarse approximation of the final result. The function-level programming style couples very well with the dataflow diagrams and visual languages, where a computation is visualized as a directed graph of data flowing between boxes representing either single operations or whole programs. As far as parallelization is concerned, we aim at (i) decomposing the computation into a set of maximally independent tasks, each of which is to be executed on a single processor, while balancing the load between the available processors, (ii) mapping different language constructs into the best corresponding parallel paradigms (e.g. function composition→streaming, construction→multithread shared memory, application→message passing of both argument and result), and (iii) capitalizing on the actual computational environment, in particular the streaming architecture of the cell/B.E. (Cell Broadband Engine) processor and the programmable graphics processing units (GPUs) of the last generation.

**Listing 1.**
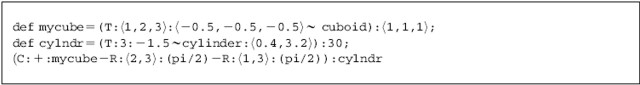
Translated cube plus translated cylinder minus two rotated cylinders.

#### (i) An example of progressive refinement

**Figure 1:**
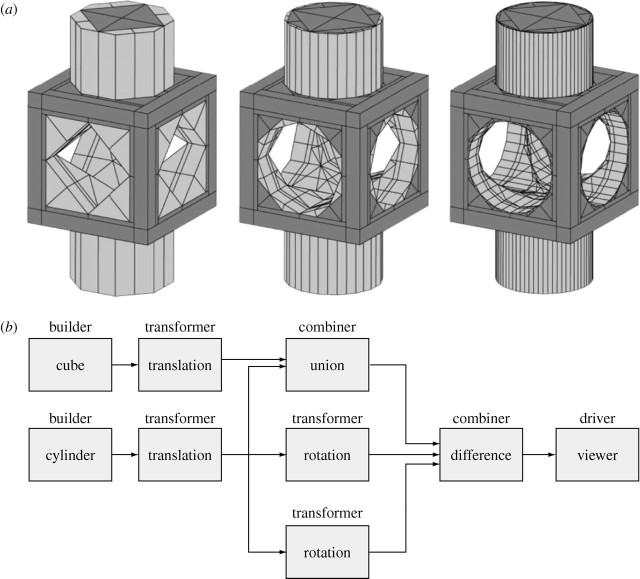
(*a*) *Progressive* geometry and (*b*) dataflow of the generating expression.

A simple example of Plasm programming is given in listing 1. If the cylndr value is generated as a stream of refinements, then the result is also progressively generated. The affine maps (rotations) R:〈2,3〉:(pi/2) and R:〈1,3〉:(pi/2) have *tensor* type, and the partial function C:+:mycube is a *curried* Boolean point-set union.^1^ The progressive generation of the geometric value produced by the evaluation of the script is shown in figure 1*a*. The dataflow diagram of the script is given in figure 1*b*.

Note, from the difference block in figure 1, that the Boolean operations are *n*-ary. In the Proto-Plasm dataflow model, a *builder* is a process with no inputs and one output stream; a *transformer* is a process with one input stream and one output stream; a *combiner* is a process with *n* input streams and one output stream; a *driver* is a process with one input stream and no output stream.

### (b) Proto-Plasm versus existing standards

Information standards are strongly needed, in order that models may be shared, evaluated and possibly developed cooperatively. In particular, standard representations enable multiple tools to be used without the need to rewrite them for each tool, and models to be published in a form that other researchers can also use in a different software environment. They also ensure the survival of models beyond the lifetime of the software used to create them (Kitano 2002).

The Systems Biology Markup Language (SBML) is a computer-readable format for representing models of biochemical reaction networks (Hucka *et al.* 2004). SBML is applicable, for example, to metabolic networks, cell-signalling pathways and regulatory networks. The development of this data language (currently arrived at level 2, v. 3) was motivated by the need of a common representation format, enabling the exchange of models between different software tools.

CellML (Lloyd *et al.* 2004; Hunter 2006; Nickerson *et al.* 2006) is an open standard based on XML, developed by the Bioengineering Institute of the University of Auckland. The purpose of CellML is to store and exchange the computer-based mathematical models of aspects and subsystems of the Physiome, possibly developed with different software tools. A CellML model consists of a number of components; each component contains a number of variables, which must be declared by placing a variable element inside the component. CellML allows for encapsulation and containment between components and enables the reuse of components from pre-existing models in a new one, inducing accelerated model building. The CellML project is closely related to FieldML (Hunter *et al.* 2006), another XML-based language. These two data languages intend to provide a vocabulary for describing the biological information at resolutions ranging from the subcellular to the organism level.

Proto-Plasm differs from the above data languages in the following two essential ways: first, Proto-Plasm is Turing complete, and hence apt to compute; second, while they work at *value* level, Proto-Plasm operates at *function* level (Backus *et al.* 1990)—making easier an automatic distribution of tasks in a concurrent environment. CellML is a data language, used to code the equations of a model. Cooper *et al.* (2006) give CellML a functional semantics, by translating it into Scheme, with translation between CellML and Scheme performed by Haskell programs. Cooper & McKeever (2007) provide a functional and referentially transparent semantics for CellML using Haskell. But no geometry description of a biomedical domain may be given by CellML, nor by its translations. Conversely, Proto-Plasm may define the model equations, but is focused on the symbolic description of model geometry and on the parallel support of simulations. Anyway, Proto-Plasm has no chance of success unless it will be interfaced with SBML and with CellML.

## 3. Basic Proto-Plasm components

The Proto-Plasm platform can be briefly described as ‘Plasm+Erlang+Hasse matrices+A-patches’, the sum standing for (i) a very high-level functional geometric language, embedded within (ii) a concurrent and distributed computational environment, using (iii) standard sparse matrices as a novel representation of the topology and geometry of the computational domain, plus (iv) piecewise cubic algebraic interpolants on *C*^1^-continuous algebraic patches, needed to map biomedical images to geometric models. In 3*a*–*f*, we briefly introduce such basic components of the platform.

### (a) Language basics

The design language Plasm is a geometry-oriented extension of a subset of FL, a functional language developed at IBM Almaden (Backus *et al.* 1990). Along the lines of his Turing Award lecture (Backus 1978), with FL Backus introduced a computational algebra in which programs are easily combined, so that new programs are obtained in a simple and elegant way. Constructed along the same lines, Plasm is extensible by design: any graphics primitive can be natively defined within Plasm and novel geometric operations can easily be added to it (Paoluzzi 2003).

#### (i) Primitive objects

The primitive objects of Plasm are: characters, numbers, truth values and *polyhedral complexes*. A polyhedron is a quasi-disjoint union of polytopes, i.e. of bounded convex sets. A polyhedral complex is a quasi-disjoint collection of polyhedra.

#### (ii) Expressions

Expressions are either primitive objects, functions, applications or sequences. According to FL semantics, an arbitrary Plasm script can be written using the following three basic programming constructs.

**Figure 2:**
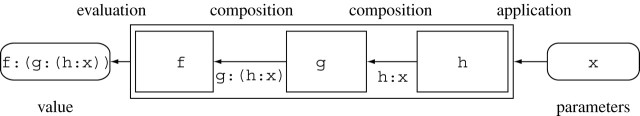
Pipelined execution: (f∼g∼h):x=(f∼g):(h:x)=f:(g:(h:x)).
- The *construction* of a vector function [f,g,h] allows for the parallel evaluation of the component functions: [f,g,h]:x=〈f:x, g:x, h:x〉 (figure 3).

- The *application* f:x of a function f to the actual value x of its input *parameter* (an element of the domain of f) yields an output *value* in the codomain of f.
- The *composition* of two or more functions produces the pipelined evaluation of their reversed sequence: (f∼g):x=f:(g:x) (figure 2).

**Figure 3:**
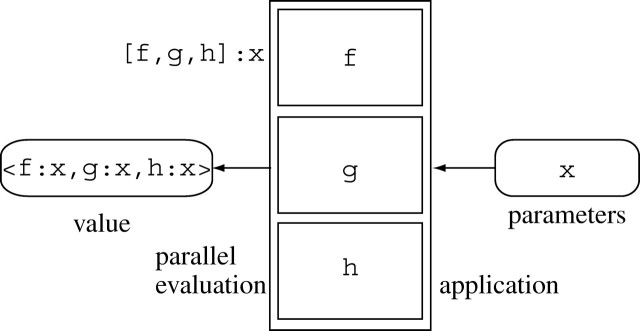
Parallel execution: [f,g,h]:x=〈f:x, g:x, h:x〉.

### (b) Basic data structures

BSP is a method for recursively subdividing a space into convex sets by hyperplanes, i.e. by affine sets of codimension 1. This subdivision gives a representation of the scene via a data structure known as *BSP tree* (Fuchs *et al.* 1980), i.e. a binary tree with partitioning hyperplanes on non-leaf nodes and with either In or Out labels on leaf nodes. A solid cell of the space partition is labelled In and an empty cell is labelled Out. A node of a BSP tree represents the convex set generated by the intersection of all the subspaces on the unique path from the node to the root. The convex set of a node equals the quasi-disjoint union of the convex sets associated with its child nodes. BSP trees are largely used in graphics, gaming and robotics. *Progressive* BSP trees, supporting *progressive* Boolean operations (Paoluzzi *et al.* 2004), are introduced in Proto-Plasm.

A *cell complex K* is a collection of *cells*, i.e. of compacts subsets of 
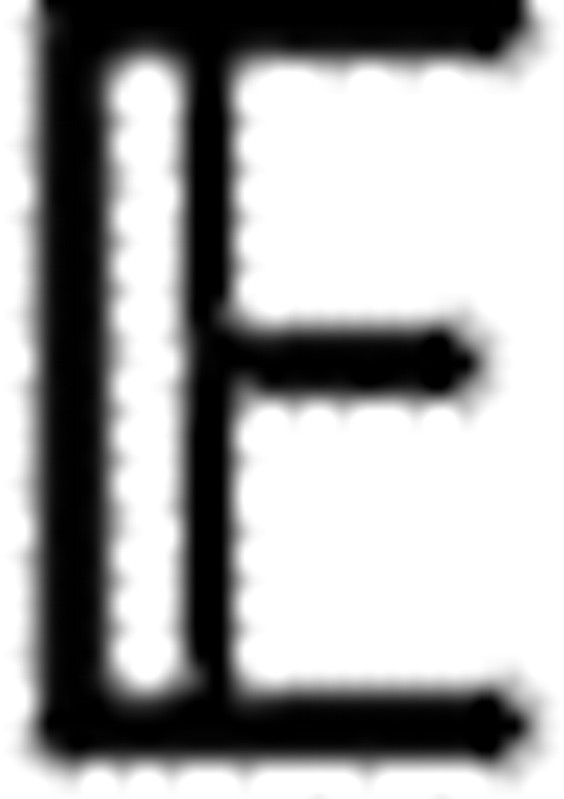
^*d*^, the *d*-dimensional Euclidean space, such that (i) if *c*∈*K*, then every *face* of *c* is in *K*; (ii) the intersection of any two cells is either empty or a face of both. A *d*-*polytope* is a solid, convex and bounded subset of 
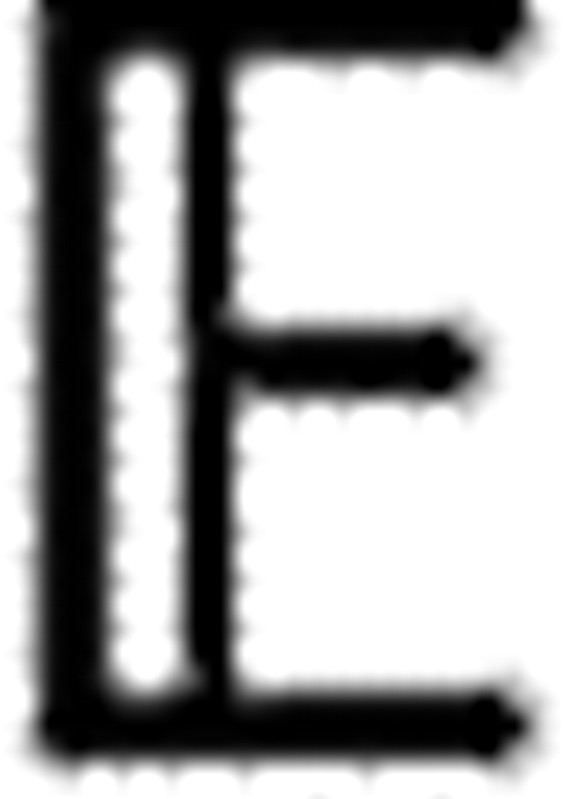
^*d*^. A *polytopal d-complex* is a cell complex of *d*-polytopes and their *k*-faces (0≤*k*≤*d*). A complete representation of a *d*-mesh is given by the *Hasse graph* of the *cover relation* of cells, whose nodes are the members of the complex *K*, partially ordered by containment, and where there is an arc from node *x* to node *y* if and only if (i) *x*⊂*y*, and (ii) there is no *z* such that *x*⊂*z*⊂*y*. Hasse graphs are currently used in the prototype language kernel as a complete representation of the topology of cell complexes, which is not handled efficiently by BSP trees.

**Figure 4:**
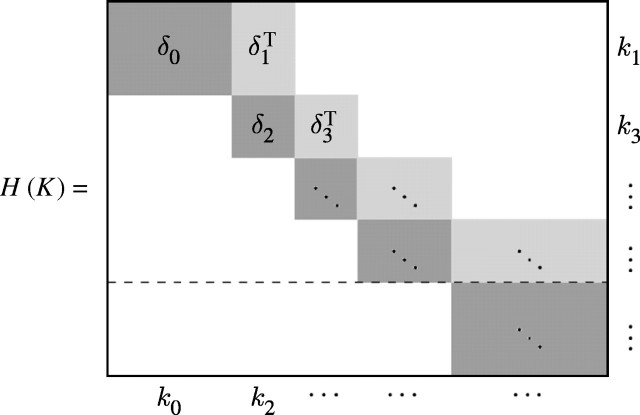
The whole scheme holds for *d* odd; for *d* even, the last block row should be discarded (*k*_*i*_ denotes the cardinality of the *k*-skeleton, i.e. the number of *k*-cells).

DiCarlo *et al.* (2007, in press) have recently shown that the (co)chain complex associated with a cell decomposition of the computational domain—i.e. with a *mesh*, as is commonly called in computational science and engineering—can be represented by a block bidiagonal matrix that we call the *Hasse matrix*, whose blocks correspond to the adjacency matrices of coboundary operators 
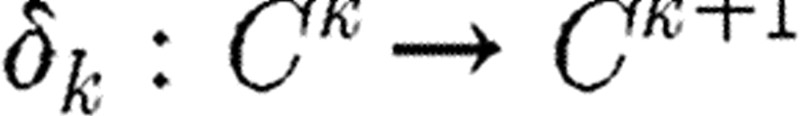
, with *C*^*k*^ the linear space of *k*-cochains associated with *k*-cells (0≤*k*≤*d*). Since the boundary operators ∂_*p*_(*p*≥1) are well represented by incidence matrices and the coboundary operators *δ*^*p*−1^ by their transposes, we may represent the *p*-families of homomorphisms ∂_*p*_, *δ*^*p*−1^(*p*≥1) by a block-structured matrix. Let *K* be a *d*-complex and 
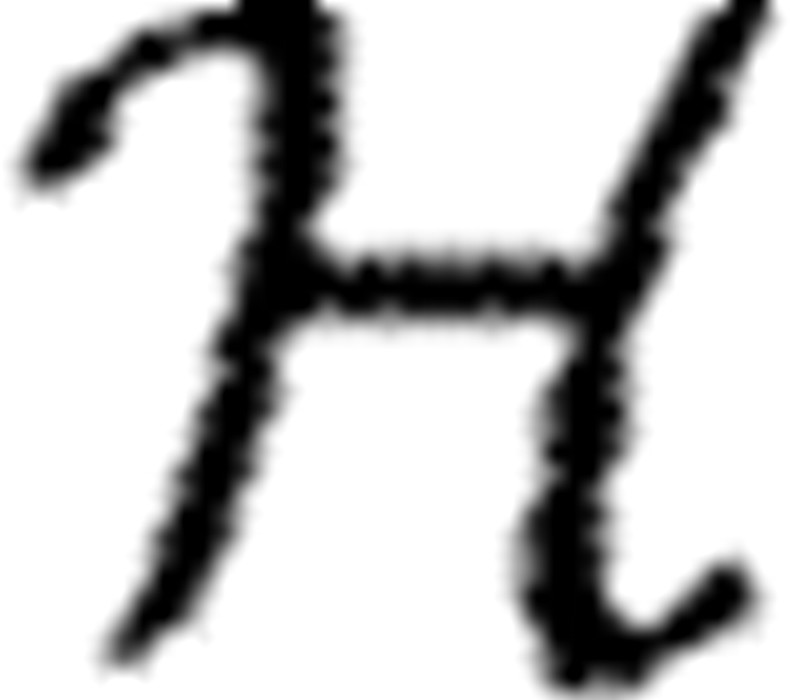
(*K*) its Hasse graph. The *Hasse matrix H*(*K*) will have the block structure shown in figure 4.

Its transpose *H*(*K*)^T^ represents the dual complex K^*^, whose Hasse graph 
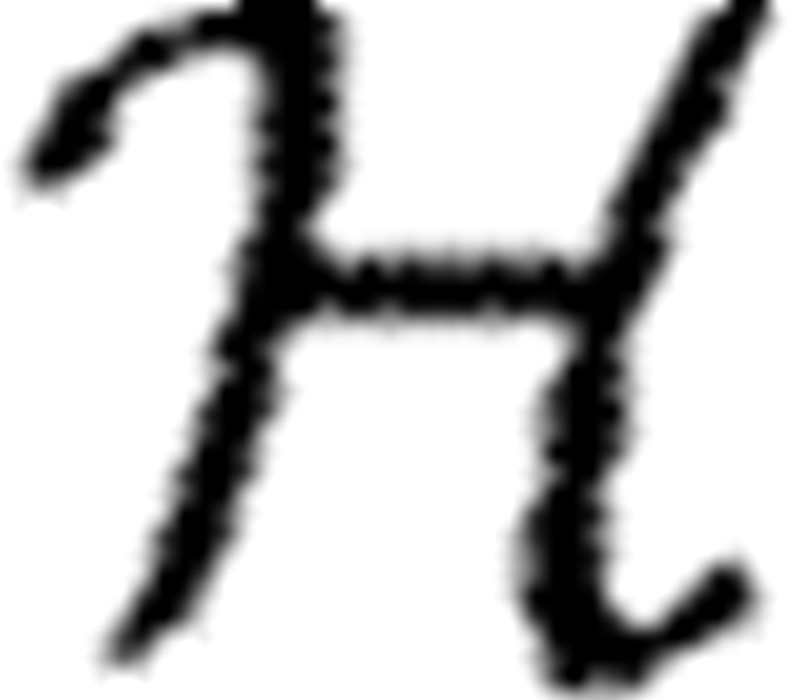
(K^*^) is isomorphic to 
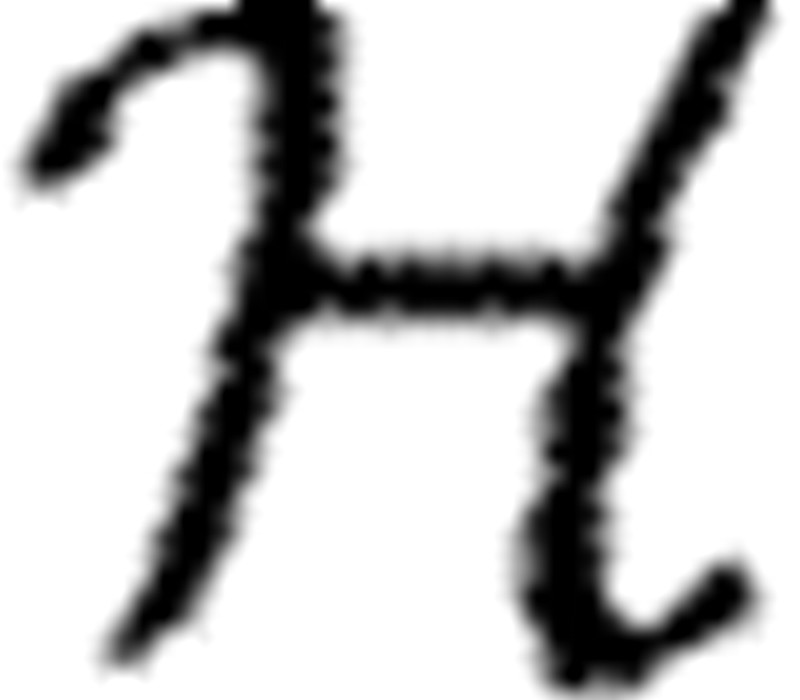
(*K*), with 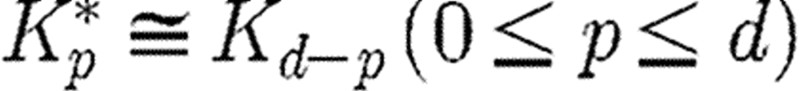, where the boundary and coboundary operators are swapped by duality. Topology-preserving mesh refinements, produced by the action of Euler operators on *K* (Mantyla 1988), can be reduced to multilinear transformations of *H*(*K*).

### (c) Algebraic representation of the computational domain

The Hasse representation of (co)chain complexes is being ported to Proto-Plasm, with the aim of simplifying and making algebraic the interface between domain representation and field computations, within an *adaptive multigrid* approach to simulation. Contrary to what might appear at first sight, the present approach does not imply higher theoretical complexity, since the number of non-zero elements in the Hasse matrix *H*(*K*) is essentially the same as the number of adjacency pointers in a typical graph-based representation of the cell complex *K*.

The (co)chain-complex formalism and the Hasse-matrix representation generalize in a natural and straightforward way to physical modelling. Chains assign measures to cells, measures that may be tuned to represent the physical properties of interest (mass, charge, conductivity, stiffness and so on). Cochains, on the other side, may be used to represent all physical quantities associated to cells via integration with respect to a measure. The coboundary operator stays behind the basic structural laws (balance and compatibility) involving physically meaningful cochains. So, a proper use of the Hasse matrix has the potential to bring geometric and physical modelling within a unified computational framework. In this respect, we wish to remark that the same data structures and algorithms—namely, linear algebra with sparse matrices—may be used for both solid modelling and physics-based simulations. From our vantage point, boundary representations and finite-element meshes appear as two different aspects of the same Hasse representation.

This approach shows promise of being a significant progress towards the close integration of geometry representations and physics-based simulation, i.e. in concurrent modelling of shape and behaviour. It should also be noted that chain complexes are a standard tool for representing and analysing topological properties of arbitrary cellular spaces. It follows that this algebraic representation may codify general models, without restrictions on orientability, (co)dimension, manifoldness, connectivity, homology and so on.

### (d) Piecewise-continuous algebraic patches

A-patches are smooth algebraic surface patch families (Bajaj *et al.* 1995), defined using a fixed degree trivariate polynomial within a compact polyhedral domain—also called the *patch scaffold*. Simple A-patches use a tetrahedron, or a cube, or a triangular prism scaffold. Prism A-patches (Bajaj & Xu 2001; Bajaj *et al.* 2002) are low-degree finite elements of algebraic surfaces, with dual implicit and rational parametric representations. Bajaj & Xu (2001) consider a matched triangulation pair 
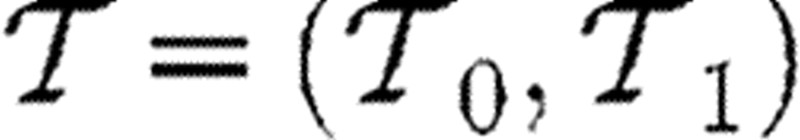
—also called a *fat triangulation*—with attached normals at each vertex, providing a piecewise linearization of the inner and outer boundary surfaces of a shell-like domain. The fat triangulation is used to reconstruct a *smooth fat surface*, whose bounding surfaces approximate 
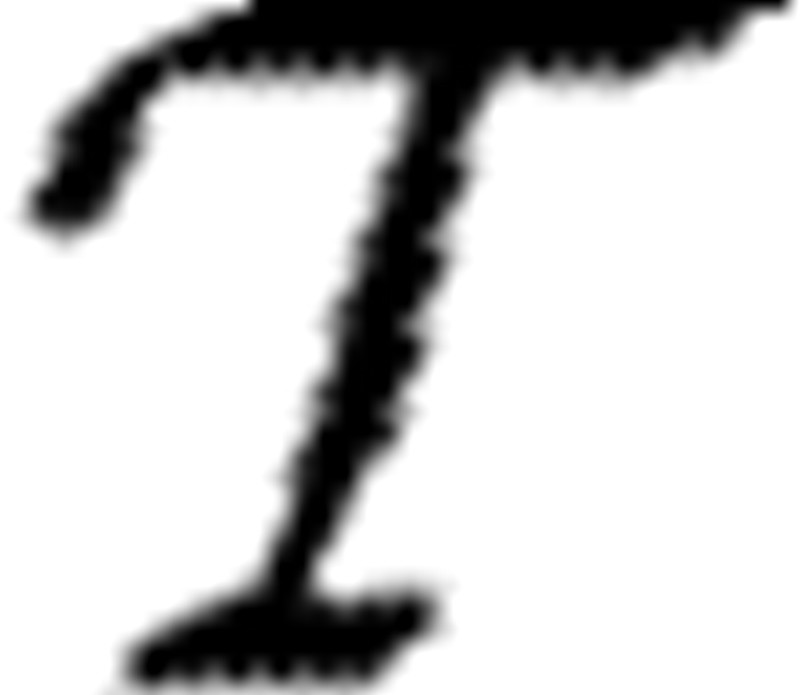
_0_ and 
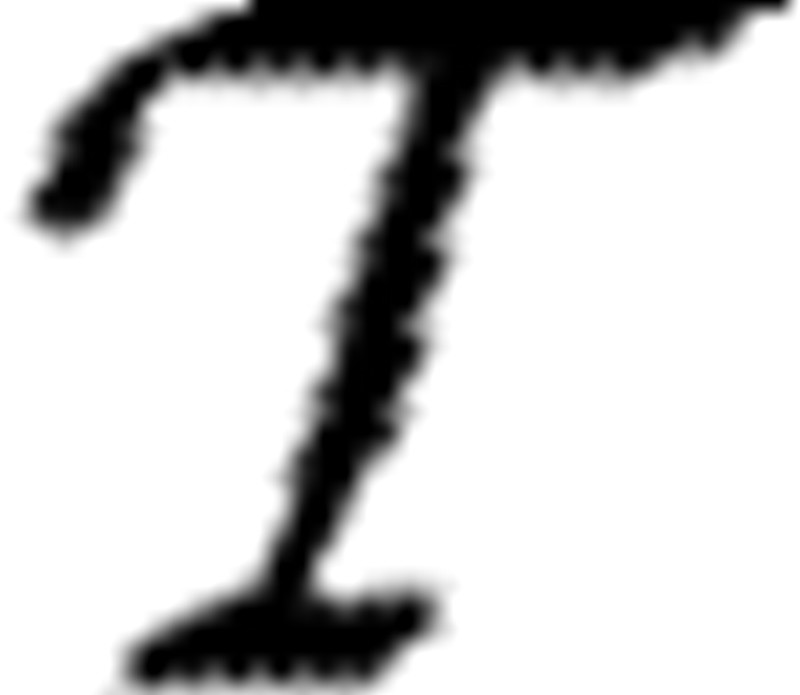
_1_, respectively. Additional intermediate surfaces, interpolating between the two boundary surfaces, may also be generated.

Matched pairs of surface triangulations can be obtained via several methods, including close isocontours of volume data and point clouds. Even a single triangulation 
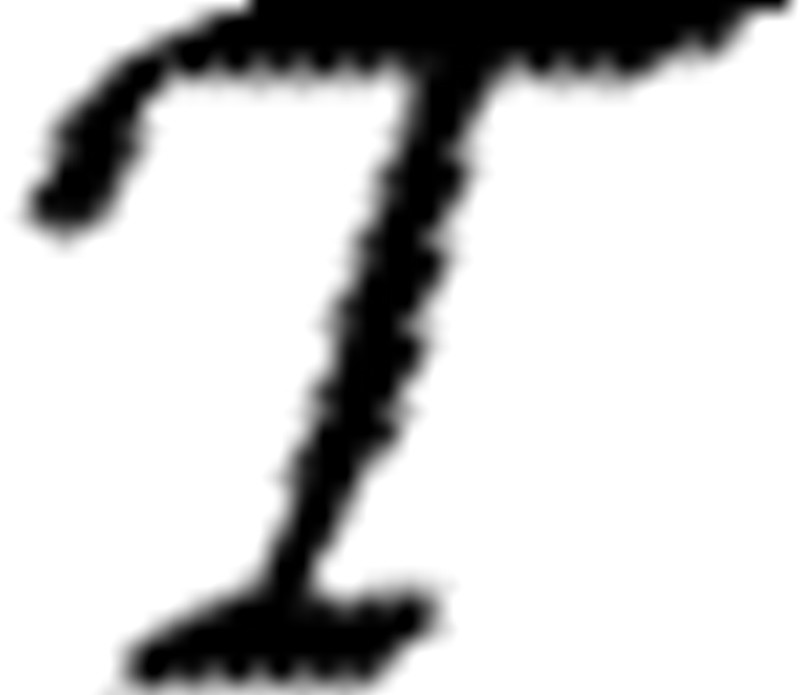
 suffices, if endowed with a set of normals, attached to either the vertices, the edges or the faces of 
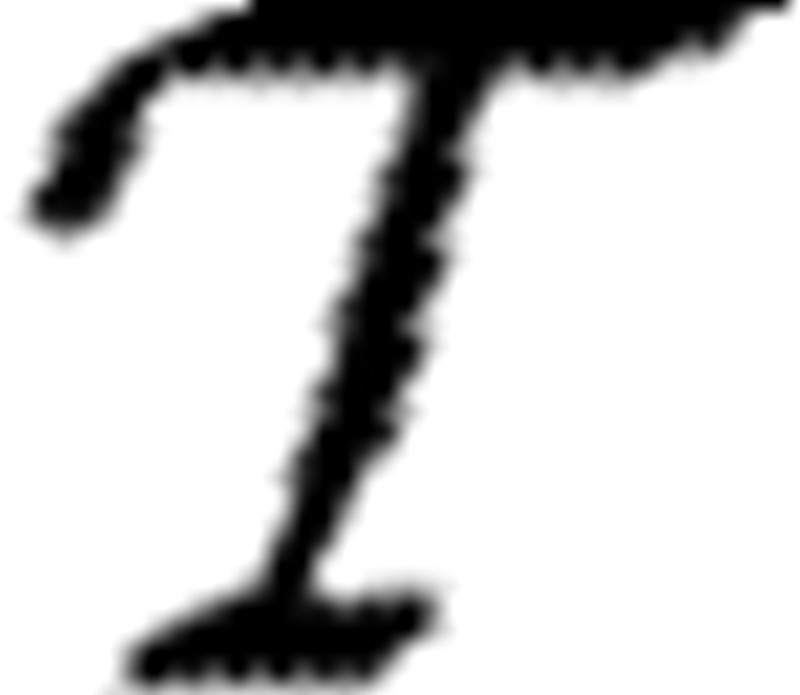
. These modelling techniques may contribute greatly to the generation of multiscale detailed meshes, driven by structural data from high-resolution tomography. Owing to the large size of these data, also mesh coarsening through surface and volumetric simplification methods is an important capability. In fact, the effectiveness of adaptive computational techniques rests on fast and robust low-level mesh primitives, including Boolean operators, for *both* refinement and coarsening.

**Figure 5:**
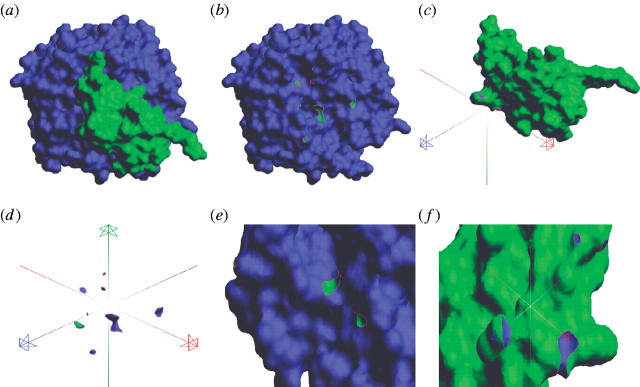
A docking pair of molecules *M*_1_ and *M*_2_: (*a*) the docked complex *M*_1_∪*M*_2_, (*b*) the difference *M*_1_\*M*_2_, (*c*) the difference *M*_2_\*M*_1_, (*d*) the intersection *M*_1_∩*M*_2_, (*e*) zoom in on *M*_1_\*M*_2_, and (*f*) zoom in on *M*_2_\*M*_1_.

Bajaj *et al.* (2008) have recently introduced a simple and robust method to trace the intersection curves between triangular or quadrilateral prismatic A-patches, while refining the support triangulation where needed. Their prototype implementation provides for the computation of Boolean operations between proteins (figure 5), offering a simple way of estimating the docking quality of very complex shapes. Further work is required to optimize the prototype software.

### (e) Concurrent and distributed computation

The C kernel of the Plasm language—named XGE (eXtreme Geometric Environment)—was recently rewritten and optimized to a large extent, in order to support very large-scale applications, such as sensor fusion for monitoring critical infrastructures (Assogna *et al.* 2008). This work has produced the Plasm v. 6.2, able to deal with meshes of approximately 200 K 3-cells in a single processing node. We are currently developing a prototype implementation, embedding the XGE geometric library into the Erlang language for concurrent computing (Armstrong 2007*a*,*b*).

Erlang is an (open source and multiplatform) concurrent functional programming language and runtime system, characterized by strict evaluation, single assignment and dynamic typing. It is purely functional, without mutable state (induced by multiple assignment), easy to understand and to debug. Type checking is performed at run-time. Erlang follows the model of computation that uses ‘actors’ as universal primitives of concurrent computing: data structures, functions, semaphores, ports, demons, processes, contexts and databases are all but special cases of actors, whose behaviour can be described by message passing.

Erlang was developed by Ericsson to support distributed, fault-tolerant, soft real-time, non-stop applications. Even hot swapping of programs is supported, so that code can be changed without stopping a system. The Erlang language seems to fit very well with multicore CPUs and SMP architectures, since processes belong to the programming language, and not to the operating system. The language has several useful features (Armstrong 2007*a*,*b*) for concurrent computation: (i) creating and destroying processes is very fast, (ii) sending messages between processes is very fast, (iii) processes behave in the same way on all operating systems, (iv) a very large numbers of processes may coexist—up to hundreds of thousands, (v) processes share no memory and are completely independent, and (vi) processes interact only through message passing.

In particular, the Erlang-based Proto-Plasm prototype translates each generating function of geometric values—hence, both the associated symbol and the tuple of formal parameters—into an Erlang process that encapsulates the chain complex and its properties within the local store, and which natively interacts via message passing.

Owing to the concurrent environment provided by the Erlang run-time system, Proto-Plasm computations will be able to scale over diverse high-performance hardware, with processors very different in type and number. Compiling a generative expression into a dataflow graph of threads is well suited to SMP machines, whereas space decomposition fits well with clusters and grids. The parallelism of Proto-Plasm modelling is based on (i) the concurrency of processes in a dataflow network fed by a continuous stream of shape refinements, (ii) the generation of model segments to be evaluated in parallel as a queue of independent jobs, and (iii) the geometric data structures of its kernel, based on BSP trees and sparse matrices.

### (f) Dataflow approach to modelling and simulation

From the very beginning of a simulation, Proto-Plasm will be able to produce a meshed approximation of the model generated by a symbolic expression, to be locally improved while new refinement cells traverse the computational pipeline. This framework, centred on the Hasse representation of the computational domain, unifies several finite formulations (Milicchio *et al.* 2008) and supports local refinement and coarsening (Scorzelli *et al.* in press).

Generative modelling matches well with the *dataflow* model of computation, which relies on a graph representation of programs, alternative to von Neumann's stored-program, one-word-at-a-time execution model. In Proto-Plasm, each operation is computed progressively by reading a stream of incremental refinements of the operands. Each refinement of the input is mapped instantly to a refinement of the output, producing a result as a stream of progressive refinements. The computation of models results in a tree of pipelined processes that make continuous progress concurrently, until the desired precision is obtained. Progressive refinements may be implemented as dataflow processing (Bajaj *et al.* 2006*a*,*b*; Scorzelli *et al.* in press) on state-of-the-art streaming hardware, such as the GPUs or the cell/B.E. processor (see Gschwind *et al.* 2006; Williams *et al.* 2006).

## 4. Simple programming examples

In this section, a toy problem in geometric programming is dealt with, in order to attest to the outstanding expressive power of a *function-level* programming style as opposed to a *value-level* approach. The powerfulness of Plasm stems from its two main features: (i) it is *dimension independent*, i.e. it treats uniformly geometrical objects of any dimension and (ii) it has native expressions for *transfinite* interpolation and approximation of *n*-manifolds (curves, surfaces, solids and higher dimensional manifolds). The term ‘transfinite’ alludes to interpolation/approximation in *infinite*-dimensional function spaces, as opposed to finite-dimensional point spaces.

### (a) How to generate a schematic heart model

Before giving the few lines of Plasm needed to produce a schematic—but parametrized—heart model, we introduce the MAP embedding operator.

#### (i) The embedding operator

The MAP operator is the constructor of curved parametric geometries. Its semantics is MAP:mapping:domain, where 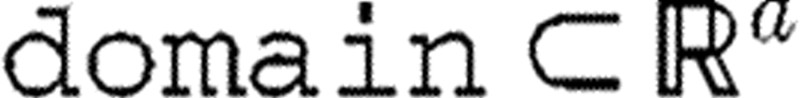, with *d*≥1 the dimension of the embedded manifolds. The mapping parameter is a sequence 〈*x*_1_, *x*_2_, …, *x*_*n*_〉 of Cartesian *coordinate functions*,
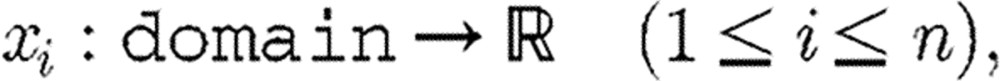
in the embedding Euclidean point space 
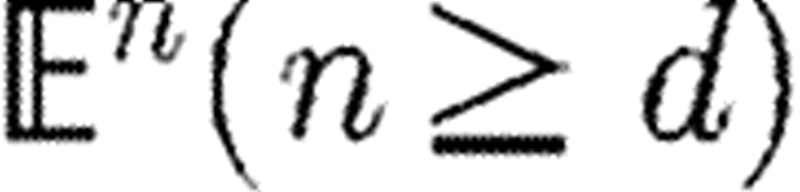
.

**Listing 2.**
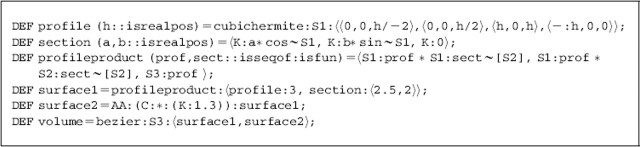
A toy heart model: definition of profile and section curves; transfinite operator profileproduct; interior surface1 and exterior surface2; volume mapping.

#### (ii) A schematic heart model

A toy heart model is generated by two curves (listing 2), denoted as profile and section, depending on one and two real parameters, respectively. Their signatures are
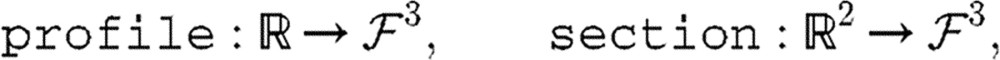
where 
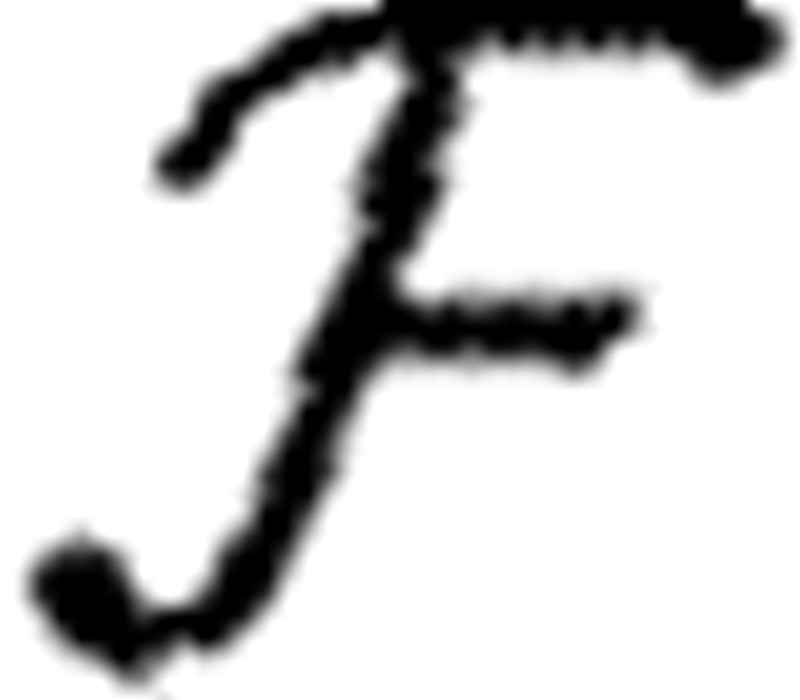
^3^ is the space of triples of coordinate functions, namely 〈*x*_1_, *x*_2_, *x*_3_〉. The profile is defined as a *cubic Hermite* curve with endpoints ***p***_0_=(0, 0, −*h*/2) and ***p***_1_=(0, 0, *h*/2), and tangent vectors ***t***_0_=(*h*, 0, *h*) at ***p***_0_ and ***t***_1_=(*h*, 0, 0) at ***p***_1_. The section curve is an ellipse with semiaxes *a, b* in the *x*_3_=0 plane, centred at the origin. The code given in listing 2 generates a family of ∞^3^ solid manifolds parametrized by three positive real numbers 
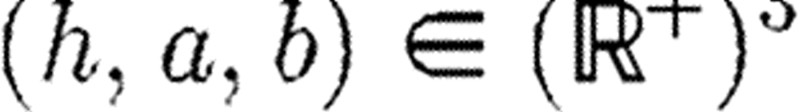
.

The profileproduct operator has signature
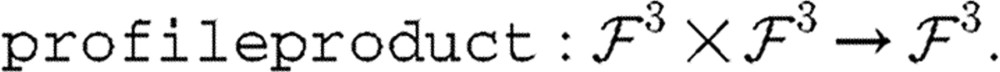


It generates the bivariate map of a surface in 
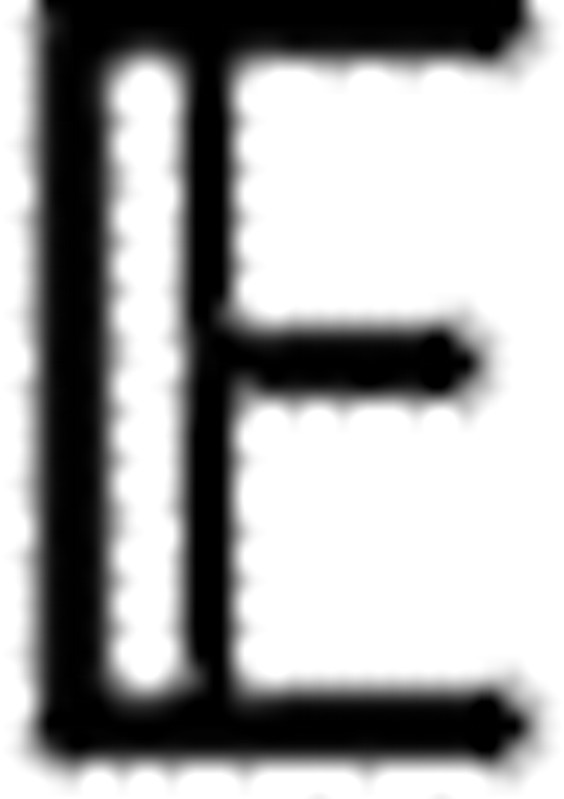
^3^, from the univariate maps of two planar curves in 
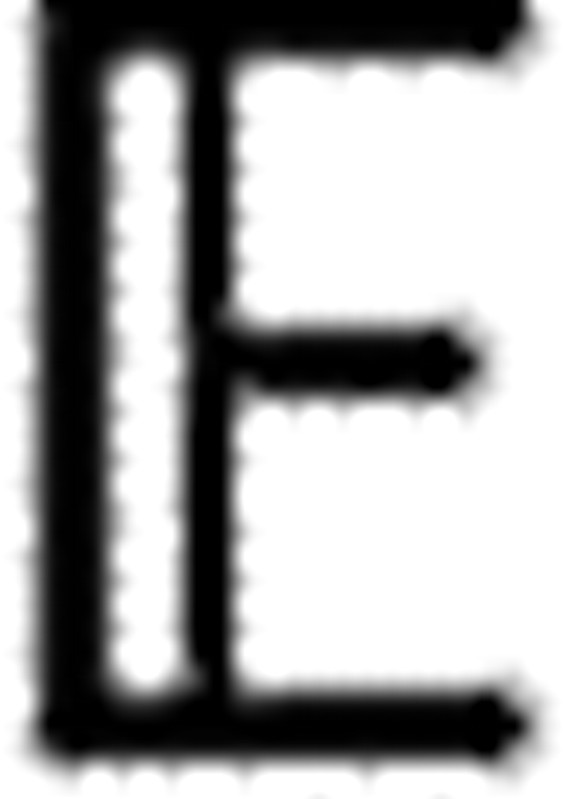
^3^, denoted prof and sect, lying in the *x*_2_=0 and *x*_3_=0 planes, respectively. In short, two curves 
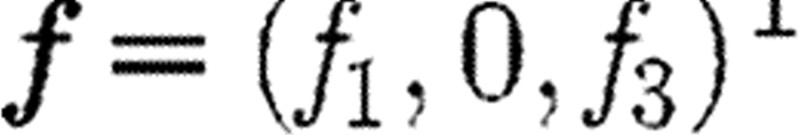
 and 
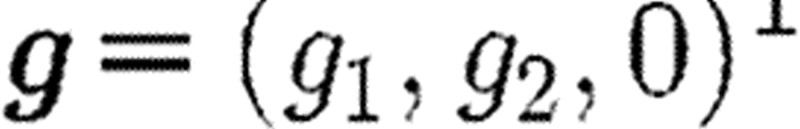
 produce a surface ***S***, to be thought of as generated by an *f*_1_-scaling plus an *f*_3_-translation of the section curve ***g***:
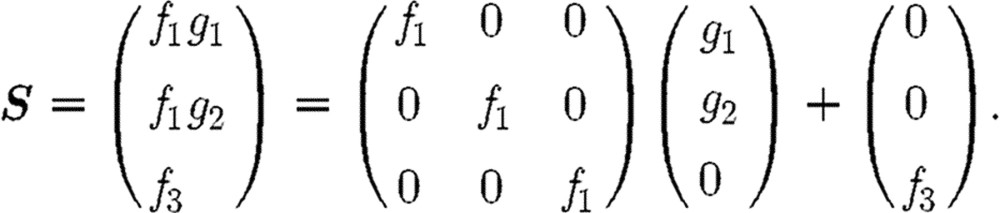


The surface2 mapping is produced by dilating surface1 by the scaling factor 1.3. The volume mapping is produced by a linear transfinite Bézier interpolation of the two surface mappings.

#### (iii) Meshing curves, surfaces and solids

**Figure 6:**
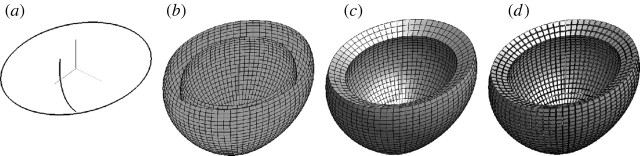
Piecewise approximation of a toy heart model. (*a*) Two generating curves (with the top removed), (*b*) interior and exterior surfaces, (*c*) solid model, and (*d*) exploded view of 3-cells.

**Figure 7:**
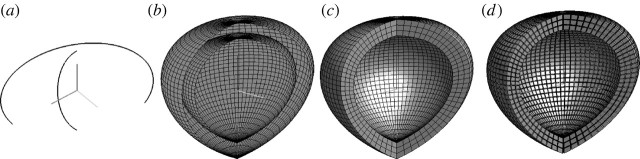
(*a*–*d*) The univariate vector mappings profile : 3 and section : 〈2.5,2〉 are evaluated here on the intervals [0, 1] and [2*π*/3, 2*π*], respectively.
- In listing 4 the *bivariate* maps surface1 and surface2 are evaluated on the rectangle [0,0,6]×[0,2*π*] subdivided into *n*×*m*=24×72 2-cells. The two resulting piecewise-bilinear approximations are shown in figure 6*b*.
- In listing 5 the *trivariate* volume mapping is evaluated on the parallelepiped [0,0,6]×[0,2*π*]×[0,1] subdivided into *n*×*m*×*p*=24×72×3 3-cells. The resulting piecewise-trilinear approximation is shown in figure 6*c*.

The entire solid mesh is generated by simply giving a profile and a section curve, that map to cell decompositions of the intervals [0,1], [0,2*π*], respectively. The portions shown in figures 6 and 7 are produced by applying the same mappings to suitable subintervals. Note that the asterisk is overloaded, denoting different products (function product, multiplication of real numbers and Cartesian product of point sets) depending on the type of its arguments.

- In listing 3 the *univariate* profile:3 mapping is evaluated on the interval [0,0,6] subdivided into *n*=24 1-cells, the mapping section:〈2.5,2〉 is evaluated on the interval [0,2*π*] subdivided into *m*=72 1-cells. The two resulting piecewise-linear approximations are presented in figure 6*a*.

### (b) Generating patient-specific heart models

**Figure 8:**
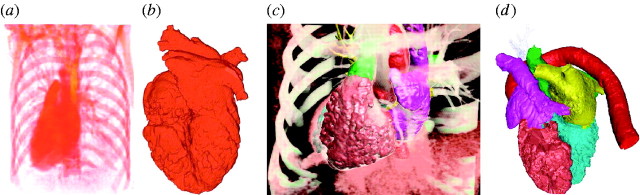
Spatially realistic finite-element reconstruction of human heart from patient-specific imaging data (Bajaj & Goswami 2008): (*a*) volume rendering from CT Angio imaging of a human thoracic region, (*b*) the initial reconstructed heart model extracted from the rest of the surrounding thoracic bone and soft tissue, (*c*) volume rendering of filtered, classified and segmented region of the thoracic volume, and (*d*) multicompartment segmented finite-element heart model, complete with the aorta and muscle walls.

Bajaj & Goswami (2008) explore a solution to the problem of constructing good finite-element and boundary-element models of the human heart from high-resolution computer tomography (CT) imaging data. In fact, the quality of patient data, even if acquired with state-of-the-art CT hardware, is not sufficient to produce satisfactorily meshed models of the anatomy and to perform further simulation for diagnostic purposes. Imaging is therefore only the initial step of a complex computational pipeline, needed to generate a robust and spatially realistic mesh model of a patient's heart (figure 8).

Rough imaging data are first put through an image processing pipeline to enhance their quality. An initial model is extracted and passed through a geometry processing, where the model is curated to remove topological anomalies, then segmented into anatomically meaningful subunits, using a symbolic template of anatomical parts, and finally meshed.

## 5. Application to complex subcellular systems

Initial applications of Proto-Plasm computational environment will mostly focus on biosystems of the living molecular cell. This modelling effort requires capturing chemical, electric and mechanical phenomena such as flexible protein–protein or flexible protein–RNA interactions in their local organelle domain—e.g. ribosomes decoding mRNA and synthesizing proteins in the endoplasmic reticulum—or self-organization and aggregation of macromolecular structures within the crowded cytosol of the cell. This section is devoted to an important modelling problem in this category, where physical information is going to be attached to an adaptive, full-dimensional decomposition of the computational domain. Giving pre-eminence to the cells of highest dimension allows one to generate the geometry and to simulate the physics simultaneously. Such a formulation removes artificial constraints on the shape of discrete elements and unifies the commonly unrelated finite methods into a single computational framework (see §3*c*).

### (a) Neuromuscular junctions

**Figure 9:**
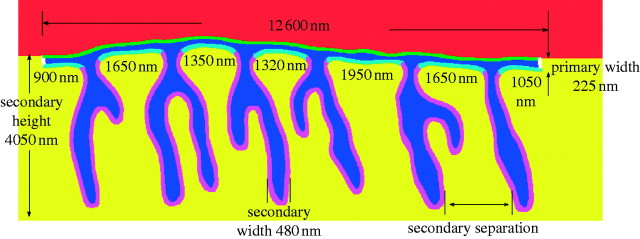
A cross-sectional unfolded layout of the interface of a typical frog NMJ, showing the cell membrane parameters to be reconstructed via spatially realistic finite-element modelling using Proto-Plasm. The red region is part of the interior of the axonal process, while the yellow region covers the interior of the muscle. The muscular postsynaptic membrane wall, densely populated with neurotransmitter receptor ion channels (AChR: acetylcholine receptors) is highly invaginated, with invagination depths reaching 4 μm and separation widths approximately 1.5 μm, as well as 0.5 μm in thickness. The pale blue and deep purple colouring signifies the two different densities of ion channels that populate the muscle membrane wall. The synaptic cleft, the region of diffusion of ACh, is coloured in dark blue, with primary width of approximately 0.25 μm.

NMJs are the points of communication between neurons and muscle fibres, transferring electrochemical signals from the neuron to the muscle and ultimately inducing the contraction of myofibrils. Figure 9 depicts a typical NMJ, featuring a relatively smooth presynaptic (neuron side) membrane and a more convoluted postsynaptic (muscle side) surface consisting of postjunctional folds and crests. Synaptic transmission is triggered by the arrival of an action potential (electric signal) to the presynaptic neuron. This action potential then triggers the release of the neurotransmitter acetylcholine (ACh) into the synaptic cleft via fusion of ACh-bearing vesicles with the presynaptic membrane. Once released, ACh diffuses throughout the intercellular space, where it interacts with two biomolecules of primary importance: the acetylcholine receptor (AChR) ion channel and acetylcholinesterase (AChE), the biomolecular ‘off switch’ for synaptic transmission via ACh hydrolysis.

**Listing 3.**
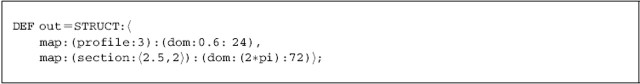
One-dimensional manifolds in figure 6a.

**Listing 4.**
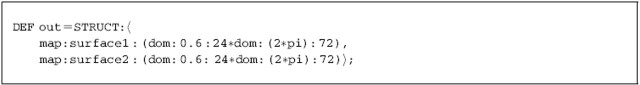
Two-dimensional manifolds in figure 6b.

**Listing 5.**
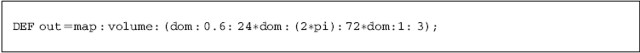
Three-dimensional manifold in figure 6c.

### (b) Multiscale modelling of the neuromuscular junction

Previous computational work in this area has either considered the molecular components of the NMJ in isolation or has used highly simplified models of the NMJ with only gross molecular information. Unlike previous published work, we aim to integrate biological length scales from molecular structure to synaptic morphology, and thereby permit the study of molecular mechanisms of anticholinergic agent action while including the broader context of the NMJ system (Zhang *et al.* 2005*a*; Cheng *et al.* 2007). The result will be a Proto-Plasm package implementing adaptive methods to solve the differential equations of continuum diffusion and electrostatics on geometries reconstructed from X-ray crystallography and NMJ morphological data from serial section high-voltage electron tomography of thick sections. This software might be used to examine the impact of a variety of biological and chemical nerve agents on the synaptic transmission for several different muscle types.

### (c) Structural modelling of neuromuscular junctions

**Figure 10:**
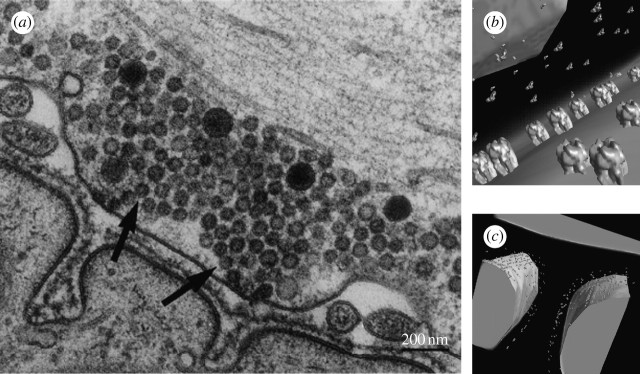
(*a*) A single slice of a transmission electron micrograph of a NMJ showing the presynaptic axonal end with many spherical vesicles filled with neurotransmitters (pointed to by arrows). Pre- and postsynaptic membranes. (Image courtesy http://www.starklab.slu.edu.) (*b*,*c*) Snapshots of a portion of our spatially realistic three-dimensional NMJ model spanning molecular and cellular scales. The AChR ion channels that populate the postsynaptic muscle membrane are shown in quasi-atomic resolution (1.5 nm), as are the AChE (enzymes) molecules floating in the synaptic cleft and attached to the basal lamina with collagen stalks (not shown).

The first step in this modelling activity is the collection of structural data for the NMJ and its biomolecular components. Given the electron microscope (three-dimensional EM) tomographic data on both fast- and slow-twitch rat soleus NMJs, the morphologies of the synapse (figures 9 and 10) are segmented and meshed (Bajaj *et al.* 2003; Zhang *et al.* 2005*a*). The location and orientation of catalytic subunits AChE within multimers in the synaptic cleft, and the location of AChR molecules on the postsynaptic membrane are approximated from pharmacological and experimental data (Zhang *et al.* 2005*a*; Cheng *et al.* 2007). All membrane data are segmented from tomographic three-dimensional EM data, and finite-element meshes are constructed using the methods of Bajaj *et al.* (2003), Bajaj & Yu (2005) and Zhang *et al.* (2005*b*). In particular, these meshes are constructed using a combination of volumetric surface feature extraction and contouring methods to discretize the space between the biomolecular surfaces and the coarse mesh obtained from the tomographic data. The second goal of this meshing is to provide a decompositive geometric description of the domain to serve as a template for placement of the more accurate biomolecular structural information. Detailed biomolecular finite-element meshes of AChE and AChR molecules are constructed by Bajaj & Yu (2005), Zhang *et al.* (2006), and Bajaj (2007) from cryo-electron microscopy data deposited in the Protein Data Bank or the European Molecular Biology Database. The molecular models of AChR are unioned into the finite-element membrane model and the AchE enzymes are unioned into the volumetric three-dimensional finite-element domain, by placement at a certain distance from the basal lamina which is intermediate in the synaptic cleft. The new algorithm for Boolean operations given in Bajaj *et al.* (2008) shall be used for this purpose. Novel progressive and adaptive refinement of the computational domain will be allowed after code optimization and integration in the new Proto-Plasm engine. A (co)chain-based formulation of the diffusion problem at different time and space scales is currently under study.

## 6. Conclusion

To stay with the applications to subcellular systems—which we privilege as the first arena where to put Proto-Plasm to the test—the computational methodology proposed in this paper may be extended to a wide variety of other systems where the key biomolecular components can be enumerated and approximate biological structural data are available.

However, our approach is by no means confined to the molecular/cell scale. Highly challenging problems, relevant to the VPH project, emerge on all scales. Our groups are active also at supracellular scales, and we plan to import such expertise into Proto-Plasm libraries. In particular, several mesoscale modelling issues have been dealt with in cardiovascular biomechanics by Nardinocchi *et al.* (2005), Nardinocchi & Teresi (2007), Tringelová *et al.* (2007) and Cherubini *et al.* (2008) and in bone remodelling by DiCarlo *et al.* (2005, 2006) and Bajaj *et al.* (2006*a*,*b*).

The computational environment provided by Proto-Plasm seems especially fit for exploring the fascinating realm of the growth and remodelling of living tissues, at scales ranging from molecular to cellular to organ scale. Adopting a top-down approach, the continuum formalism developed by DiCarlo & Quiligotti (2002) and DiCarlo (2005) makes it possible to test quantitatively the predictions of coarse, but coherent and workable, models against macroscopic physiological data, often largely available. When properly tuned, these models would offer a well-structured target to bottom-up efforts to bridge the gap between molecular machinery and physiological function.

## Footnotes

Given a function *f* : *X*×*Y*→*Z*, currying it gives the function Curry *f* : *X*→{*Y*→*Z*} such that (Curry *f*)(*x*): .
